# NLRP3 downregulation enhances engraftment and functionality of adipose-derived stem cells to alleviate erectile dysfunction in diabetic rats

**DOI:** 10.3389/fendo.2022.913296

**Published:** 2022-07-22

**Authors:** Chao Luo, Yaqian Peng, Xiongcai Zhou, Junhong Fan, Weihong Chen, Haibo Zhang, Anyang Wei

**Affiliations:** ^1^ Department of Urology, Nanfang Hospital, Southern Medical University, Guangzhou, China; ^2^ Key Laboratory of Endemic and Ethnic Diseases, Ministry of Education and Key Laboratory of Medical Molecular Biology of Guizhou Province, Guizhou Medical University, Guiyang, China; ^3^ Department of Urology, Guangzhou Eighth People’s Hospital, Guangzhou Medical University, Guangzhou, China; ^4^ Department of Urology, Guangdong Provincial People’s Hospital, Guangdong Academy of Medical Sciences, Guangzhou, China

**Keywords:** adipose derived stem cell, erectile dysfunction, pyroptosis, gene modification, diabetes mellitus

## Abstract

**Background:**

The transplantation of adipose-derived stem cells (ASCs) is a most promising treatment for diabetic erectile dysfunction (DMED). However, the effect of high glucose on the post-transplantation survival of stem cells limits the efficacy of ASCs transplantation. Prolonging the survival time of ASCs *in vivo* after transplantation is a key issue in the utilization of ASCs for DMED. Herein, we aimed to investigate the therapeutic effect of ASCs by downregulating NOD-, LRR-, and pyrin domain-containing protein 3 (NLRP3) as well as its mechanism of action in DMED.

**Methods:**

ASCs were obtained by isolating subcutaneous fat from SD rats and were identified using lipogenic and osteogenic differentiation assays, as well as flow cytometric analysis. The shNLRP3 lentivirus with the best downregulating effect was screened, and shNLRP3 lentivirus (LV-shNLRP3) was transfected into ASCs (ASCs^shNLRP3^) to detect apoptosis and the reactive oxygen species (ROS) levels in each group under high glucose conditions. In DMED rats, ASCs^LV-shNLRP3^, ASCs^LV-control^, or phosphate buffered saline (PBS) were administrated *via* intra-cavernous injection, and normal rats served as normal controls. One week post-injection, animal imaging was performed to track the ASCs. Four weeks post-injection, erectile function was evaluated by measuring the intra-cavernosal pressure and mean arterial pressure. Corpus cavernosum pyroptosis and endothelial function were examined by western blotting and immunofluorescence.

**Results:**

NLRP3-mediated pyroptosis might be a pathogenic mechanism of ED and DMED. ASCs were isolated successfully. Thereafter, the LV-shNLRP3 with the highest transfection efficiency was selected and used to modify ASCs successfully. LV-shNLRP3 could protect ASCs paracrine function under hyperglycemia through anti-apoptosis and anti-ROS deposition mechanisms. Furthermore, ASCs^LV-shNLRP3^ showed an advantage in the suppression of pyroptosis compared to ASCs^LV-control^. The ASCs^LV-shNLRP3^ group had improved cavernous endothelial function and smooth muscle injury, thus reversing erectile function, and was superior to the ASCs^LV-control^ group.

**Conclusions:**

NLRP3 Inflammasome-mediated pyroptosis might be involved in DMED formation. Intra-cavernous injection of ASCs^LV-shNLRP3^ could suppress cavernosal pyroptosis, contributing to improved erectile function in DMED rats.

## Introduction

Nearly 35–90% of diabetic patients have varying degrees of erectile dysfunction (ED) (1). Men with diabetes are 1.9-4 times more likely to experience ED and on average experience ED 15 years earlier than men without diabetes (2–4). The current first-line treatment for diabetic erectile dysfunction (DMED) involves phosphodiesterase-5 inhibitors (PDE5Is), however, PDE5Is can be ineffective in 30–40% of the population, most patients have to opt for penile prosthesis surgery, which has significant postoperative complications including pain. Therefore, it is important to explore new therapeutic approaches and new pathophysiological mechanisms for DMED.

The erectile tissue is a specialized vascular tissue structure, composed of small resistance arteries and spiral arteries, which lead to a sinusoidal cavity arranged *via* a single-layer vascular endothelium. The vascular endothelium is usually covered by the network structure composed of smooth muscle cells, extracellular matrix, and autonomic nerves. In addition, collagen, elastic fibers, and fibroblasts form part of the extracellular matrix. The interaction between these tissue structures results in the hemodynamic and mechanical process of penile erection, which is very important to the whole erectile system (5, 6). The pathogenesis of DMED is complex, but ultimately both can lead to endothelial dysfunction and impaired smooth muscle relaxation, either of which might affect erectile function.

Adipose-derived stem cells (ASCs) therapy is considered a promising treatment for DMED (7). Its advantages include a high number of ASCs per volume of subcutaneous adipose tissue, a high rate of proliferation; additionally, anti-fibrotic, anti-apoptotic, immunomodulatory, anti-inflammatory, and paracrine mechanisms have been demonstrated in preclinical studies (8–11). Angiogenic growth factors (e.g., vascular endothelial growth factor [VEGF] and insulin-like growth factor-1 [IGF-1]) secreted by ASCs are present in body tissues. These proteins tend to induce proliferation and angiogenesis and can protect against vascular ED (12). Furthermore, ASCs function in reducing autophagy to treat diabetic nephropathy and inflammation by secreting exosomes and *via* differentiation (13, 14). However, the erectile-protective function of ASCs in treating DMED rats has not yielded satisfactory results and has not yet been applied in clinical work (15). One of the main reasons is that stem cell transplantation is difficult and short-lived in a high glucose and inflammatory environment. Interestingly, Czech (16) deleted the heat production inhibitory gene *NRIP1* through CRISPR and efficiently depleted its product in human ASCs *in vitro*; compared to that with unmodified adipocyte implantation, implanting this CRISPR-enhanced human or mouse brown-like adipocytes into mice fed a high-fat diet was found to reduce obesity and liver triglycerides and improve glucose tolerance. Xu (10) found that HIF1α overexpression could enhance diabetic wound closure in high glucose and low oxygen conditions by promoting adipose-derived stem cell paracrine functions and survival. Furthermore, some studies found that HIF-1α, VEGF, and GDNF modify diverse stem cells to enhance their paracrine function and promote their curative effect in the treatment of ED (17, 18). In summary, the knockdown or overexpression of some key genes in ASCs will increase the response of stem cells in a high glucose and hypoxia environment.

As one of the main complications of diabetes, DMED is closely associated with inflammation. Of related markers, the NLRP3 inflammasome is the most well-characterized multimeric protein complex to date (19). NLRP3 inflammasome-mediated inflammatory cytokines can act in both an autocrine and a paracrine manner and contribute to multiple chronic inflammatory diseases and metabolic disorders, such as obesity, hypertension, diabetes, atherosclerosis, and cancer (20–24). These diseases also have a close relationship with dysregulation of the endothelium, by altering both active participants and regulators of inflammatory processes (24). Thus, targeting NLRP3 has great therapeutic benefits (25). Furthermore, most studies on ED have been performed at the animal level, and whether the mechanisms identified in these studies can be applied to human studies remains debatable. Accordingly, this study explored the relationship between ED and pyroptosis through bioinformatic analysis techniques. We then hypothesized that the knockdown of NLRP3 (ASCs^LV-shNLRP3^) in ASCs could promote longer cell survival in a high glucose environment and alleviate the erectile dysfunction in DMED rats *via* paracrine secretion. In this study, we tested this hypothesis using DMED rats and found that NLRP3 downregulation could enhance erectile functions in diabetic rat by promoting ASCs paracrine functions and survival.

## Materials and methods

### Bioinformatic analysis

According to our previous study (26), differentially expressed genes (DEGs) between the ED and non-ED group were screened from the Gene Expression Omnibus dataset using the ‘limma’ package of R (4.0.0). Pyroptosis-related genes were obtained from GeneCard (https://www.genecards.org/). Briefly, we entered the keyword “pyroptosis”, and downloaded the data in the format of “txt.” The DEGs and pyroptosis-related genes were then entered into the Venn diagram (http://bioinformatics.psb.ugent.be/webtools/Venn/) to obtain pyroptosis-related differential expressed genes (PRGs). Next, we mapped the PRGs to the KEGG database using ‘ClusterProfiler’ and ‘org.Hs.eg.db’ packages of R software to identify associated pathways. The cut-off values were set according to the parameters of an absolute logFC >0.5 and false discovery rate < 0.05.

### Isolation, culture, and identification of ASCs

Primary ASCs were obtained as described previously (27). Fat tissue of Sprague-Dawley (SD) rats weighing 180–220 g was isolated from the inguinal area, minced in digestive solution containing 0.15% type I collagenase, and incubated under shaking at 120 rpm in a constant-temperature hybridization oven (UVP, Upland, CA, USA) at, 37°C for 1 h. Subsequently, adipose tissue was resuspended in 10% FBS (HyClone, GE, Boston, MA,USA) complete medium to terminate the digestion and washed with PBS by centrifugation at 1200 rpm, then processed with FACS Lysing Solution (BDIS Catalog No.349202) for 10 min. The final precipitate was collected and cultured in stem cell culture medium (CM-R198, Procell, Wuhan, China). Cells from the third passage were used for adipogenic and osteogenic induction using the corresponding inducing medium (PanEra laboratories. Inc., Beijing, China) for 21 days to confirm the multipotential differentiation capability. The cell surface markers of ASCs were measured using a FACScan flow cytometer (BD, Franklin Lakes, NJ, USA) using fluorochrome-conjugated antibodies, including CD29-FITC (1 µg/test; 11-0291-82, eBiosciences, CA), CD45-APC (0.5 μg/test; 17-0461-82, eBiosciences, CA,USA), and CD90-PE (0.06 μg/test; 12-0900-81, eBiosciences).

### Lipogenesis assessment and oil red O staining experiment

ASCs with good growth status at the P2 passage were selected and spread in 6-well plates to induce the cells grow to cover 70% of the bottom of the plate, or approximately 1.5 × 10^5^ cells/well. According to the instructions of the lipogenesis induction Kit (Abcam, Cambridge, UK), we performed adipogenesis induction with ASCs. The induced growth of the cells was observed daily under an inverted microscope and photographed and recorded. When the lipid-inducing solution was administered for 7–14 days, ASCs appeared as many round vesicle-like lipid droplets. Induction was terminated when the number of lipid droplets was high or when induction reached 21 days. Next, according to the manufacturer’s instructions, we prepared oil red O dye in advance (Abcam, Cambridge, UK) and stained the induced cells. Then, the culture dishes were moved into the inverted microscope (Olympus, x63, Japan) to observe and photograph them.

### Osteogenesis and alizarin red staining experiment

ASCs with a good growth status at the P2 passage were selected and spread in 6-well plates to induce the cells grow to cover 70% of the bottom of the plate, or approximately 1.5 × 10^5^ cells/well. The osteogenic induction of ASCs was performed using the Osteogenic Induction Kit (Santa Cruz Biotechnology, Santa Cruz, CA, USA). In general, when osteogenesis was induced for approximately 7–14 days, more obvious round calcified nodules gradually appeared on the surfaces of ASCs, and the number of calcified nodules gradually increased with time. When many calcified spots appeared or when induction reached 21 days, induction was stopped as appropriate. Then we prepared Alizarin red staining solution (Santa Cruz Biotechnology) in advance and stained the induced cells. Afterwards, the culture dishes were moved into the inverted microscope (Olympus, x63, Japan) to observe and photograph them.

### Lentiviral-shNLRP3 construction and transfection

A specific LV-shNLRP3 (a lentiviral LV-control carrying luciferase and NLRP3 knockdown interfering plasmids) was designed by JiKai Gene Company (Shanghai, China) and verified by sequencing. The carrier number was GV344, and the original order was hU6-MCS-Ubiquitin-firefly_Luciferase-IRES-puromycin. Three LV-shNLRP3 lentiviral LV-controls were constructed based on their original order. The sequence of the NLRP3 interference plasmid is presented in **Table 1**. The lentivirus supernatant was screened and used for multiplicity of infection (MOI = 50). Three dishes of cells were each transfected with one of three lentiviruses to screen the best lentivirus. Puromycin was used to screen for stable cells. The dishes were maintained in a humidified atmosphere containing 5% CO2 and 95% air at 37°C. The medium was changed 8 hours after lentivirus transfection and then every 24 hours. After 72 hours, cells were harvested to detect *NLRP3* mRNA and protein expression using quantitative real-time PCR (qRT-PCR) and western blot (WB) assays. After their third Passage, the transfected cells were used for experiments. To explore the function of ASCs transfected with NLRP3 knockdown lentivirus (ASCs^LV-shNLRP3^). ASCs were divided into three groups: ASCs, ASCs transfected with vector lentivirus (ASCs^LV-control^) and ASCs^LV-shNLRP3^, that were cultured in a high glucose medium (30mmol/L) for 24 hour.

**Table 1:** The sequence of NLRP3 interference plasmid.

ID	5’	stem	loop	stem	3’
Nlrp3-RNAi (92977-1)-a	CCGG	GTGGATAGGTTT GCTGGGATA	CTCGAG	TATCCCAGCAAA CCTATCCAC	TTTTTG
Nlrp3-RNAi (92977-1)-b	AATTCA AAAA	GTGGATAGGTTT GCTGGGATA	CTCGAG	TATCCCAGCAAA CCTATCCAC	_
Nlrp3-RNAi (92978-1)-a	CCGG	CCGAAAGAAGT TGCTGCCTAA	CTCGAG	TTAGGCAGCAAC TTCTTTCGG	TTTTTG
Nlrp3-RNAi (92978-1)-b	AATTCA AAAA	CCGAAAGAAGT TGCTGCCTAA	CTCGAG	TTAGGCAGCAAC TTCTTTCGG	_
Nlrp3-RNAi (92979-1)-a	CCGG	AGCATCCAAGCA AGCAGGAAA	CTCGAG	TTTCCTGCTTGC TTGGATGCT	TTTTTG
Nlrp3-RNAi (92979-1)-b	AATTCA AAAA	AGCATCCAAGCA AGCAGGAAA	CTCGAG	TTTCCTGCTTGC TTGGATGCT	_

### Quantitative real-time PCR

Total RNA was extracted from the cultured cells using AG RNAex Pro Reagent (AG21101, Accurate Biology, Changsha, China) following the manufacturer’s protocols. Reverse transcription of RNA was performed by using 1 µg of RNA per 20 µl of reaction buffer with the PrimeScript RT reagent kit (Takara Bio Inc., Otsu, Japan). qRT-PCR was performed using LightCycler^®^480 II (Roche, Basel, Switzerland) with the SYBR Green PCR kit (Takara, Japan) according to the manufacturer’s protocol. qRT-PCR amplification with specific primer sets for NLRP3, VEGFA, SDF-1, FGF2, and β-actin was conducted at an annealing temperature of 55–60°C for 40 cycles. The following primers were used: NLRP3, forward, 5′-CAGAAGCTGGGGTTGGTGAA-3′ and reverse, 5′-CCCATGTCTCCAAGGGCATT-3′; VEGFA, forward, 5′-ACAGGGAAGACAATGGGA-3′ and reverse, 5′-CTGGAAGTGAGCCAACG-3′; SDF-1, forward, 5′-CCTCTGTCACCAGCCTTT-3′ and reverse, 5′-CTGCACTTCCTTCCCACT-3′; FGF2, forward, 5′-ACTTCGCTTCCCGCACT-3′ and reverse primer, 5′-GTGGGTCGCTCTTCTCC-3′; β-actin, forward, 5′-GATCAAGATCATTGCTCCTCCTG-3′ and reverse, 5′-AGGGTGTAAAACGCAGCTCA-3′. β-actin was used as the internal reference; relative expression was determined using the 2^−ΔΔCT^ method.

### Flow cytometry

The apoptosis rate was evaluated using the Annexin V-FITC/PI Apoptosis Detection kit. The cells were seeded into 6-well tissue culture plates (5 × 10^5^ cells/well). Following treatment, the cells were collected, washed with PBS, and resuspended in 500 μL binding buffer. Then, 5 μL Annexin V-FITC and 5 μL propidium iodide (PI) were added to the buffer and incubated at room temperature (PI) for 15 min in the dark. Stained cells were analyzed using a FACScan flow cytometer (BD).

### Reactive oxygen species assay

The ROS assay kit (E004-1-1, Nanjing Jiancheng Bioengineering Institute) was used for analysis. Briefly, after high glucose stimulation, ASCs in each group were washed three times with fresh PBS. Serum-free medium containing 10 μmol L^−1^ DCFH-DA was added to the culture plate. Then, an appropriate amount of the mixture was added and incubated in a cell incubator at 37°C for 30 min and then washed with fresh PBS buffer three times. ROS detection was observed by IX73 fluorescence microscopy (Olympus, Tokyo, Japan), and the appropriate area was selected for imaging.

### Establishment of a DMED rat model and ASCs implantation *in vivo*

Male SD rats (approximately 200 g and 8-weeks-old) with normal erectile function were provided by the Experimental Animal Center of Southern Medical University. The rats with DMED were modeled and identified as previously described (27). Briefly, 80 healthy adult male SD rats were intraperitoneally injected with 1% streptozotocin solution (65 mg kg^−1^). Diabetes was confirmed by measuring random blood glucose levels 72 h after injection. Rats with random blood glucose concentrations >16.7 mmol L^−1^ were diagnosed as diabetic. Eight weeks after STZ injection, an apomorphine (Sigma-Aldrich) test (100 μg kg^−1^) was carried out to confirm DMED in rats as previously described and 42 rats met the standard for ED (28). The rats with DMED were anesthetized with pentobarbital sodium (50 mg kg^−1^, i.p.) and received a bilateral intracavernous injection of 1 × 10^6^ ASCs^LV-control^ in 200 μl of phosphate-buffered saline (ASCs^LV-control^-treated group) or 1 × 10^6^ ASCs transfected with LV-shNLRP3 (ASCs^LV-shNLRP3^-treated group) (n =10 per group). Another 10 normal rats served as the control group. A successful DMED rat model should have an intracavernosal pressure (ICP) of <60 mmHg, and an ICP/MAP (mean arterial pressure) ratio of <0.5. The penis was harvested for histological and WB analyses.

### *In vivo* small animal imaging

*In vivo* tracking of LV-shNLRP3-transfected ASCs was performed using the IVIS Lumina II system, as previously described (27). Briefly, all groups of animals were intraperitoneally injected with 150 mg kg^−1^ D-luciferin (Bioworld, Minneapolis, MN, USA) dissolved in DPBS (HyClone) at a concentration of 15 mg ml^−1^ for 5 min before anesthesia. The animals were then placed in the camera apparatus, and local images were taken.

### Immunofluorescent staining

Corpus cavernosum tissues were fixed overnight in 4% paraformaldehyde. Paraffin-embedded tissue specimens were routinely prepared and sectioned at a 5 μm thickness. After antigen repair, goat serum was applied at RT for 30 min. The caspase1 antibody (1:50; 22915-1-AP, Proteintech), NLRP3 antibody (1:50; 19771-1-AP, Proteintech), α-SMA antibody (1:100; SC-53015, Santa Cruz), and CD31 antibody (1: 100; ab222783, Abcam) were incubated with the sample overnight, and goat anti-rabbit IgG (HRP) (Abcam, ab205718) was added through a drip. We used DAPI to stain the cell nuclei, and images were captured using a laser confocal microscope (Nikon, Tokyo, Japan).

### Histology and immunohistochemistry

Tissue slides were prepared similarly to those used for immunofluorescence staining. H&E, Masson’s trichrome staining, and immunohistochemistry (IHC) were performed. Sections were cut at 5 μm thickness and incubated with an anti-NLRP3 antibody (1:50; 19771-1-AP, Proteintech). Digital images were acquired with an Olympus microscope (BX63), and the smooth muscle (SM)-to-collagen ratio based on Masson’s trichrome staining was evaluated using Image-Pro Plus 8.0.

### WB assay

WB analysis was performed as described previously (27). Briefly, cellular proteins were extracted using RIPA lysis buffer (UW0103, Ubio, Shanghai, China) and centrifuged at 4°C for 20 min (15 000 rpm). Tissue proteins were fixed in liquid nitrogen and ground with a grinding rod; this process was repeated 3-5 times. Next, RIPA lysis buffer was added, shaken for 10 seconds, further ground with a tissue grinder (condition: 65 Hz, 60 seconds, 5 cycles) (Tissuelyser-32L, Jingxin, Shanghai, China), and centrifuged at 4°C for 20 minutes (15,000 rpm). Protein concentration was measured using a BCA kit (Thermo Fisher Scientific Inc., Waltham, MA, USA). The proteins were separated using sodium dodecyl sulfate-polyacrylamide gel electrophoresis (SDS-PAGE) and transferred onto polyvinylidene fluoride (PVDF) membranes. After blocking with 5% bovine serum albumin solution, membranes were incubated overnight with primary antibodies (19771-1-AP; Proteintech) at 4 °C.

The primary antibody, anti-NLRP3, was used to identify the effect of the LV-shNLRP3 blockade. β-Actin (ABclonal, AC026) was used as a loading control. The primary antibodies for cytokines included anti-VEGFA (1:1000; ab214424, Abcam), anti-SDF-1 (1:500; 17402-1-AP, Proteintech), and anti-FGF2 (1:1000; DF6038, Affinity).

Furthermore, ASC (apoptosis-associated speckled protein containing CARD) is the central adapter for NLRP3 inflammasome formation (29). The primary antibodies for pyroptosis related markers included anti-ASC (1:500; YT0365, ImmunoWay, TX, USA), anti-caspase1 (1:500; 22915-1-AP, Proteintech), anti-NLRP3 (1: 500; 19771-1-AP, Proteintech), anti-IL-1β (1:500; A16288, ABclonal), anti-IL-18 (1:500; 10663-1-AP, Proteintech), and anti-GSDMD (1:500; 20770-1-AP, Proteintech). The primary antibodies for endothelial markers included anti-CD31 (1:2000; ab222783, Abcam), anti-eNOS (1:500; ab76198, Abcam), and anti-phospho-eNOS (S1177) (1:1000; ab215717, Abcam). Anti-α-SMA (1:1000; SC-53015, Santa Cruz) was used to evaluate the cavernous smooth muscle, which is defined as the effector organ for ED (30). Furthermore, β-Tubulin (1:15000, 66240-1-lg; Proteintech) antibody was used as the loading control. Secondary antibodies were obtained from Abbkine Inc. (catalog # A21010, # A21020, USA) and diluted to 1:30,000.

### Statistical analysis

Statistical results are expressed as mean ± standard deviation (SD). The Unpaired t-test were performed for comparisons between two groups using GraphPad Prism 8. Two-tailed P<0.05 was considered statistically significant.

## Results

### Induction of NLRP3-mediated pyroptosis in cavernous tissue in ED patients

**Figure 1:**
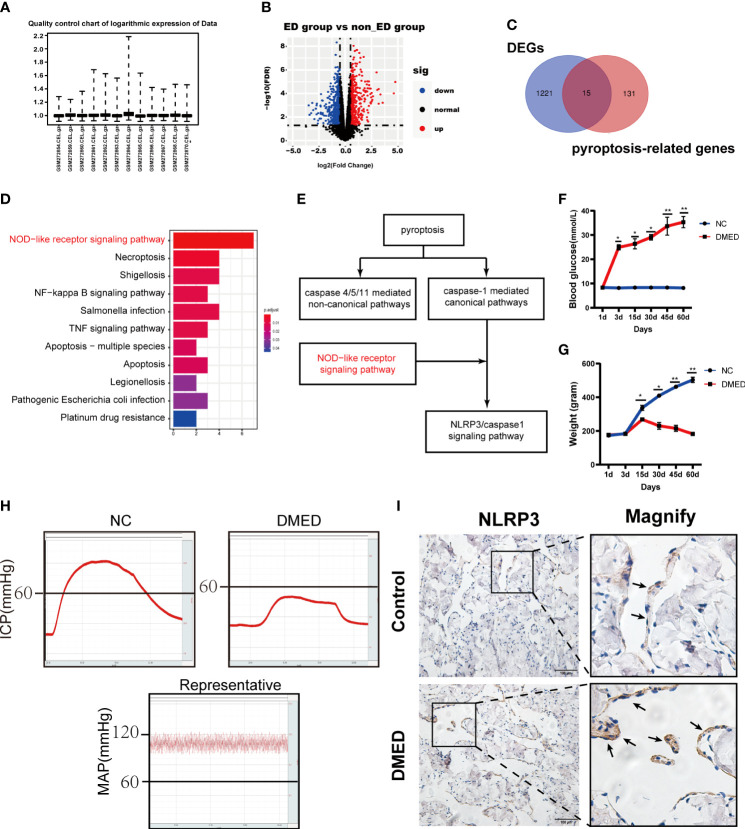
NLRP3 mediated pyroptosis may be associated with DMED. **(A)** Boxplot of the relative logarithmic expression (RLE) reflected the consistency of parallel experiments. **(B)** Volcano plots showed the differentially expressed genes (DEGs) from GSE10804. Data points in red represent down-regulated, and green represent up-regulated genes. **(C)** Venn diagram was performed to screen the PRGs from the DEGs and pyroptosis-related genes. **(D)** KEGG pathway analysis of ARGs was performed with bar plot. **(E)** NLRP3/CASP1 signal pathway was the classic pathway of two pathways of pyroptosis. **(F)** Bodyweight after 8 weeks’ feeding and **(G)** blood glucose level in each group. Each bar represents mean ± SEM. **(H)** Results of erectile function expressed as ICP and the ratio of ICP/MAP. **(I)** Immunohistochemistry staining showed the expression level of NLRP3 in corpus cavernosum between DMED and NC rat. (ns: p>0.05. * p<0.05. ** p< 0.01).

Based on a previous study, the raw data were normalized for further analysis (**Figure 1A**). Then, 1,236 significant DEGs between the ED and non-ED samples were identified. Among these DEGs, 423 genes were upregulated in ED tissue compared with levels in non-ED tissue; the other 815 genes were downregulated based on the volcano plot (**Figure 1B**). We matched 1236 DEGs with 146 pyroptosis-related genes from GeneCard **(** **Supplemental File 1** **)** using a Venn diagram, resulting in 15 PRGs **(** **Figure 1C** **)**. The results of KEGG enrichment analysis showed that PRGs were significantly enriched in the NOD-like receptor (NLR) signaling pathway **(** **Figure 1D** **)**. Caspase-1-activating NLRs have been commonly studied to date. NLR recognition of bacterial, viral, and host molecules and toxic foreign products can lead to caspase-1 activation. The NLR protein NLRP3 responds to multiple stimuli (31). Importantly, the NLRP3/caspase1 signaling pathway has been studied as a typical NLR signaling pathway in pyroptosis (**Figure 1E**) (32). Next, a DMED rat model was constructed to determine the expression levels of NLRP3. Blood glucose and animal weights were also measured and compared between the DMED and control group (NC) (**Figures 1F, G**). ICP and MAP were measured to identify the DMED and NC rats (**Figure 1H**). In the immunohistochemical analysis of the penile tissues, the expression of NLRP3 in the DMED group was higher than that in the control group **(** **Figures 1C, D** **)**. Taken together, NLRP3 inflammatory vesicle-associated pyroptosis might be involved in the formation of DMED.

### Characterization and LV-shNLRP3 transfection in ASCs

**Figure 2:**
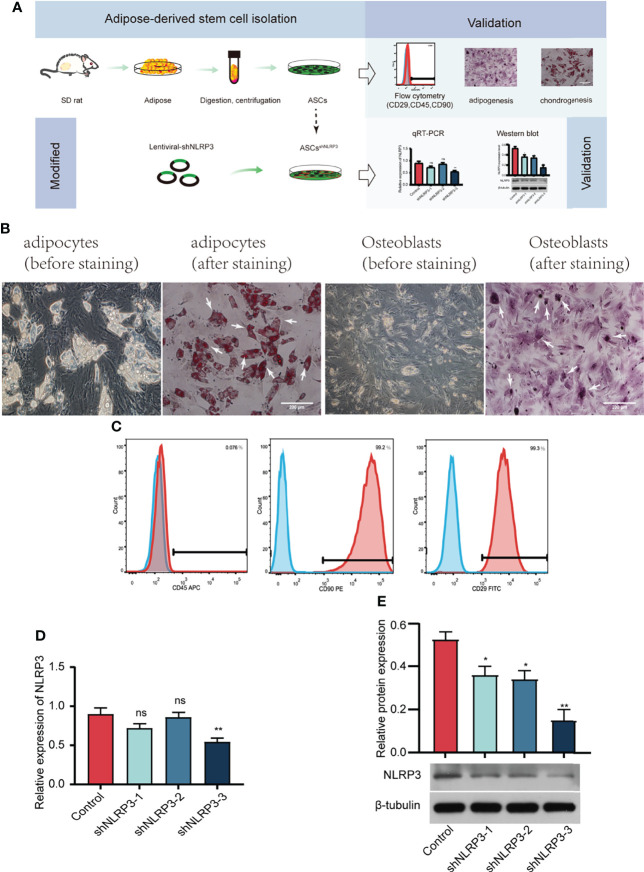
Identification of ASCs, and screening of LV-shNLRP3. **(A)** Flow chart of extraction, identification and lentiviral transfection of ASCs. **(B)** Typical cell image (left), adipogenesis and osteogenesis of ASCs confirmed by oil red O (left) and alizarin red (right) staining under × 200 magnification. (White arrows represent typical lipid droplets and calcium nodules, respectively). **(C)** Flow cytometric analysis of surface markers suggested that ASCs were positive for well-known stem cell markers, including CD29 and CD90, but not for endothelial or hematopoietic markers CD45. **(D)** qRT-PCR showed that the third of three strains of LV-shNLRP3 lentivirus were screened in ASCs. **(E)** WB showed the third of three strains of LV-shnlrp3 lentivirus were screened in ASCs. (ns: p>0.05. * p<0.05. ** p< 0.01).

We extracted subcutaneous adipose tissue from the groin area of SD rats, cleaned the blood vessels in the adipose tissue, cut, centrifuged, filtered the tissue, and then, placed it in a culture bottle **(** **Figure 2A** **)**. Adipogenesis and osteogenesis were confirmed by oil red O and alizarin red staining **(** **Figure 2B** **)**. ASCs were identified based on specific surface antigens using flow cytometry. As shown in **Figure 2C**, CD29 and CD90 were expressed in 99% of the cells, whereas CD45 was not expressed. Moreover, qRT-PCR and WB assays were performed to measure the decrease in the expression of the mRNA and protein levels of NLRP3 **(** **Figures 2D, E** **)**, indicating that the *NLRP3* gene was successfully knocked down in ASCs. Taken together, ASCs were isolated successfully, and shNLRP3-modified ASCs were prepared.

### LV-shNLRP3 transfection prolongs the survival of ASCs and promotes their paracrine function

**Figure 3:**
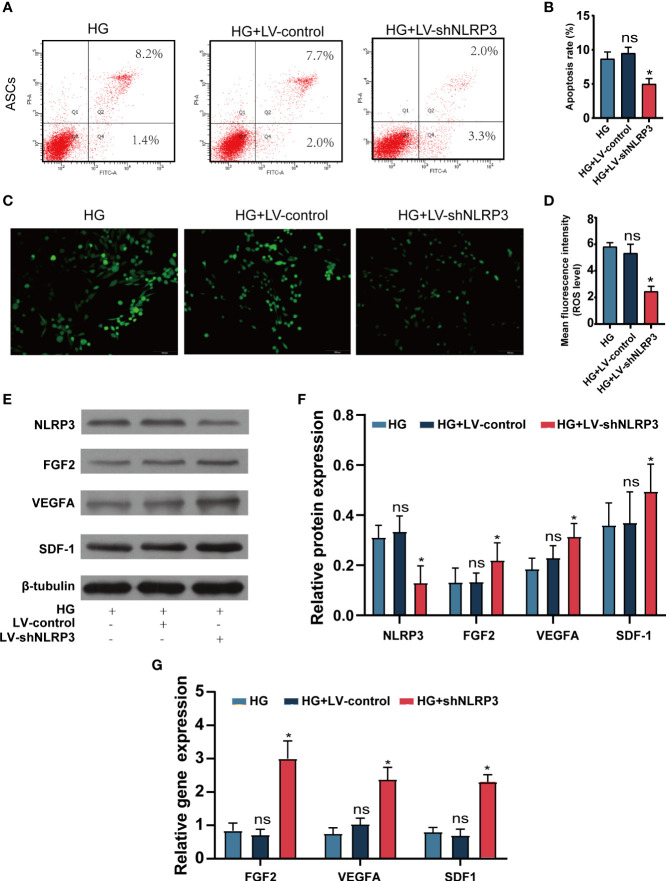
LV-shNLRP3 prolong the survival and the paracrine function of ASCs. **(A, B).** Flow cytometry was performed to check the apoptosis rate of ASCs^LV-shNLRP3^, ASCs^LV-control^, and ASCs under normal and high glucose respectively. **(C, D)** IF showed the fluorescence intensity of three kind of ASCs. (The mean fluorescence intensity represents intracellular ROS level). **(E)** WB showed the protein level of NLRP3, SDF-1, VEGFA and FGF2 in the three groups; **(F)** relative statistical analysis. **(G)** qPR-PCR showed the gene expression of SDF-1, VEGFA and FGF2 in the three groups respectively. (ns: p>0.05. * p<0.05. ** p< 0.01).

We assessed the functional characteristics of ASCs^LV-shNLRP3^ in comparison to those of ASCs and ASCs^LV-control^. Flow cytometry was performed to check the apoptosis rate of these three ASCs types under high glucose and showed that ASCs^LV-shNLRP3^ had a lower apoptosis rate than ASCs and ASCs^LV-control^ **(** **Figures 3A, B** **)**. Meanwhile, the ROS levels were measured to determine their ability to resist oxidative stress (OS). The results showed that the fluorescence intensity of ASCs^LV-shNLRP3^ was weaker than that of the other two groups **(** **Figures 3C, D** **)**, which suggested that LV-shNLRP3 enhanced the ability of ASCs to resist OS. In addition, ASCs exert their therapeutic effects mainly through paracrine cytokines (33). Accordingly, three angiogenic factors, SDF-1 (34), VEGFA (35), and FGF2 (36), were used to measure the paracrine function of ASCs. Protein levels and gene expression of SDF-1, VEGFA, and FGF2 in ASCs^LV-shNLRP3^ were higher than those in the other two groups **(** **Figures 3E-G** **)**. These results suggest that LV-shNLRP3 can protect the paracrine function of ASCs under hyperglycemia through anti-apoptosis and anti-ROS deposition mechanisms.

### LV-shNLRP3 enhances ASCs to ameliorate erectile dysfunction in DMED rats

**Figure 4:**
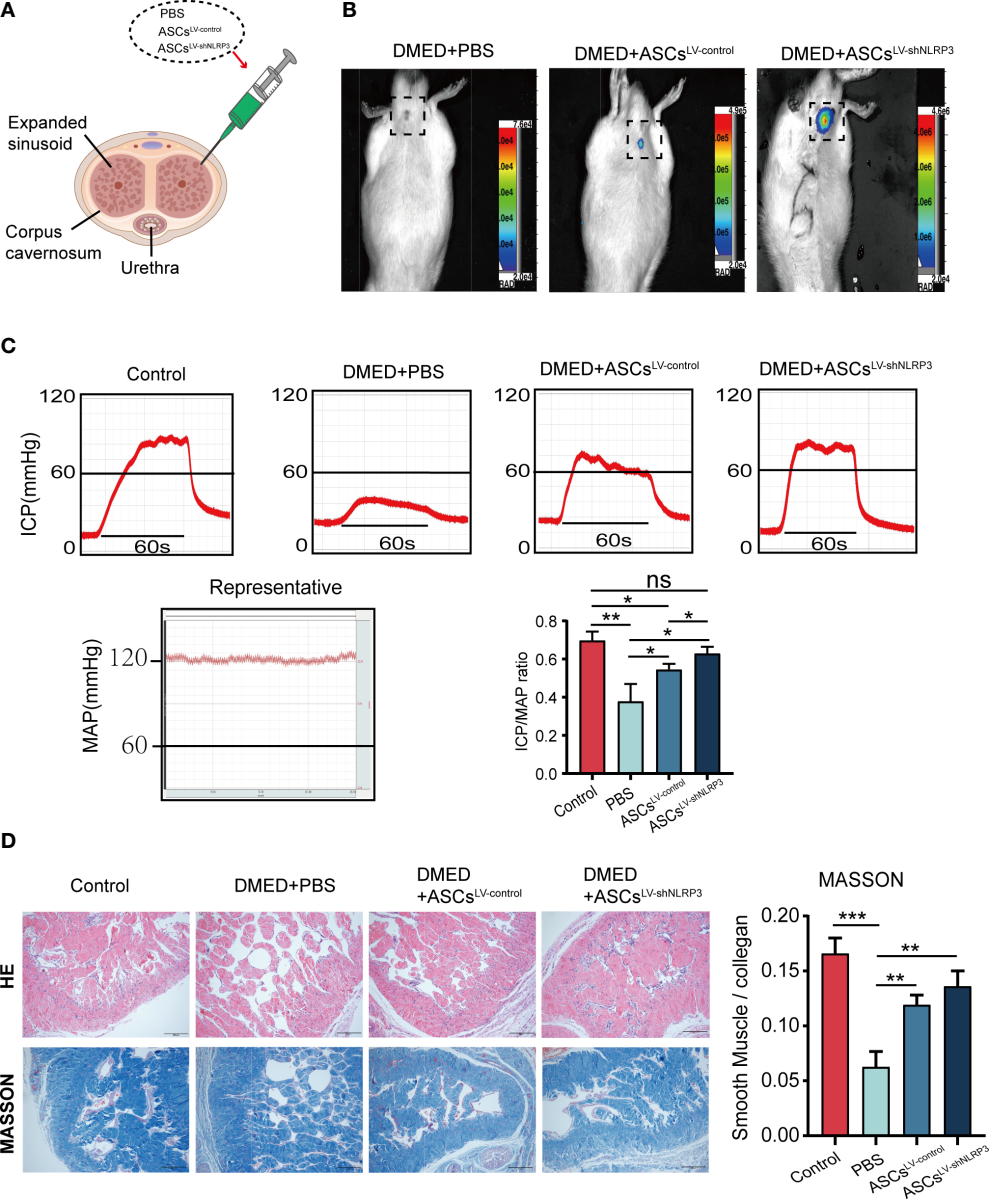
Transplantation of ASCs^LV-shNLRP3^ improved erectile function of the diabetic ED rats. **(A)** Schematic diagram of injection site of ASCs. **(B)** ASCs^LV-shNLRP3^ can prolong the survival time in cavernous tissue. **(C)** Representative images of intracavernosal pressure (ICP) and mean arterial pressure (MAP) and the ICP/MAP ratio are shown in each group of rats. **(D)** A thinner smooth muscle layer and discontinuous and disordered cavernous sinuses were found in all diabetic rats, with more severe changes in the PBS group. In the Masson’s trichrome staining images, the ASCs and ASCs^LV-shNLRP3^ groups retained the SM-to-collagen ratio compared with the PBS group, which is shown in the statistical graph. (ns: p>0.05. * p<0.05. ** p< 0.01).

To identify the function of LV-shNLRP3-modified ASCs, we injected the cavernous sinus of rats with phosphate buffered saline (PBS), ASCs, and ASCs^LV-shNLRP3^ (**Figure 4A**). After the 7th day, no fluorescence was detected in the DMED+PBS group, a control for luciferase gene transfection. The fluorescence intensities in the other two groups showed that fluorescence in the DMED+ASCs^LV-shNLRP3^ rats was notably stronger than that in the DMED+ASCs^LV-control^ rats **(** **Figure 4B** **)**. The ICP/MAP values reflected the erectile function with an equal load in the electrically stimulated cavernous nerve. Generally, the penis in the ASCs^LV-shNLRP3^ group exhibited an ICP/MAP ratio similar to that in the control group, and the penis in the DMED group showed a much lower ICP/MAP ratio than that in the control group. Specifically, the ICP/MAP ratio in the ASC^LV-shNLRP3^ group at month 4 was similar to that of the control group, and the ICP/MAP values were higher than those in the other groups **(** **Figure 4C** **)**. These results suggested that the ASCs^LV-shNLRP3^ had a positive effect on erectile function. Additionally, HE staining showed that the structure of the corpus cavernosum was irregularly arranged in the DMED+PBS group compared with that in the controls. The structure in the DMED+ASC, and DMED+ASCs^LV-shNLRP3^ groups was similar to that of the control group. Masson staining showed that the penis in the DMED+PBS group had a much lower SM/collagen ratio than that in the control group. Specifically, the SM/collagen ratio of the ASCs^LV-shNLRP3^ group at month 4 was similar to that in the control group, and SM/collagen values were higher than those in the DMED+PBS and DMED+ASCs ^LV-control^ groups **(** **Figure 4D** **)**.

### ASCs^LV-shNLRP3^ suppress NLRP3-mediated pyroptosis of the corpus cavernosum

**Figure 5:**
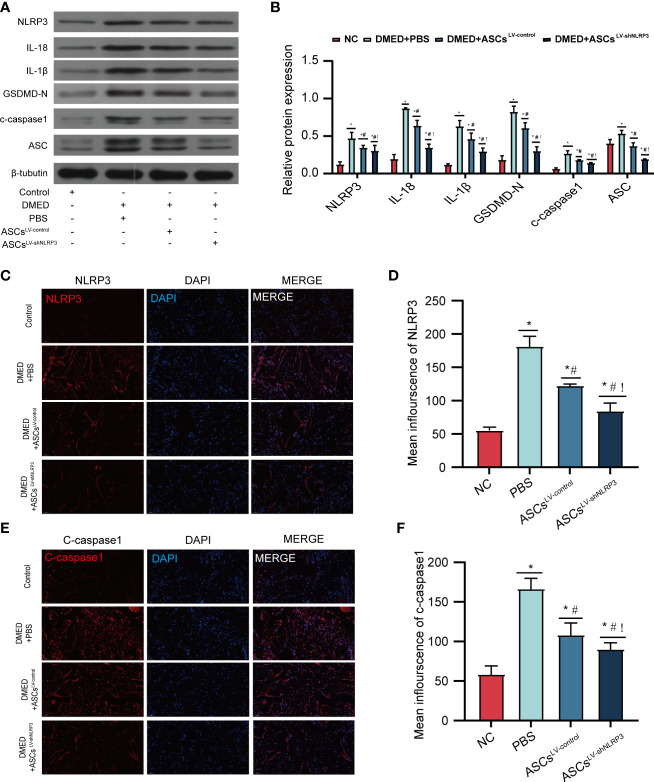
ASCs^LV-shNLRP3^ ameliorate Corpus cavernosum pyroptosis. **(A)** WB showed the protein expression level of pyroptosis-related moleculars among four groups, and **(B)** statistical analysis. **(C-F)** IF showed the fluorescence intensity of caspase-1, NLRP3 among four groups, and statistic analysis. (ns: p>0.05. *: compared with control group, p<0.05. #: compared with DMED+PBS group, p< 0.05;!: compared with DMED+ASCs^LV-control^, P<0.05^)^.

To identify the protective function of ASCs^LV-shNLRP3^ in the corpus cavernosum *in vivo*, this study was performed using DMED rats. Protein levels of pyroptosis-related markers (IL-1β, IL-18, GSDMD-N, c-caspase1, ASC) were higher in the DMED group than in the control group. Protein levels of pyroptosis-related markers decreased after treatment with ASCs ^LV-control^ and ASCs^LV-shNLRP3^ **(** **Figures 5A, B** **)**. Consistent with the protein expression, the immunofluorescence intensity of c-caspase1 and NLRP3 suggested the same conclusion **(** **Figures 5C–F** **)**. indicating that ASCs have the ability suppress pyroptosis. Furthermore, compared to ASCs^LV-control^, ASCs^LV-shNLRP3^ showed an advantage in the suppression of pyroptosis.

### ASCs^LV-shNLRP3^ reverse the corpus cavernosum endothelial function in DMED rats

**Figure 6:**
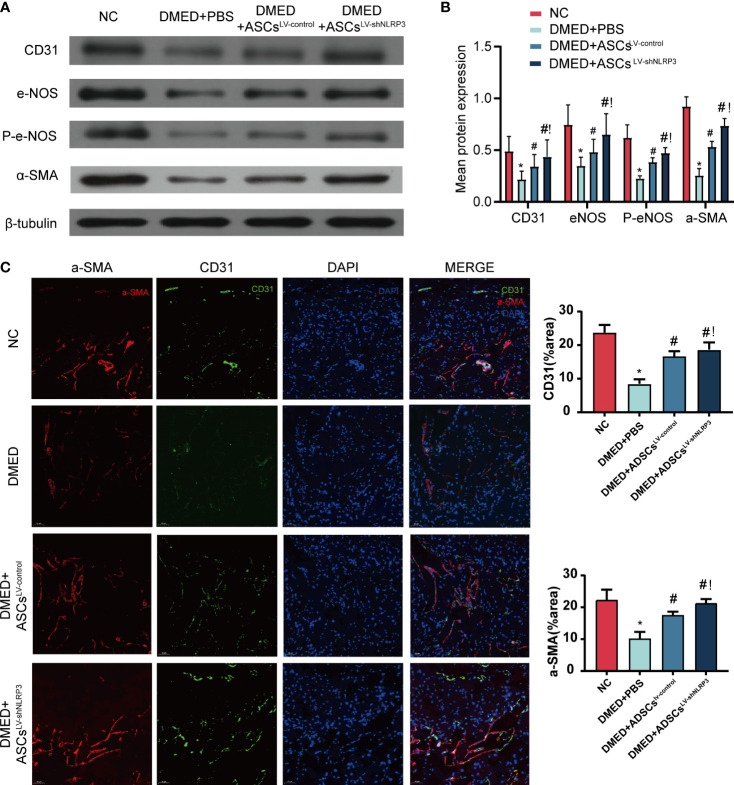
ASCs^LV-shNLRP3^ retained the endothelial and smooth muscle ingredient of cavernous body. **(A)** WB, and **(B)** quantification for WB revealed that CD31, eNOS, p-eNOS, α-SMA expression were all increased in the ASCs-treated and ASCs^LV-shNLRP3^ -treated group 4 weeks after cytotherapy. **(C)** IF showed the fluorescence intensity of CD31 and α-SMA in cavernous tissue of each group. (ns: p>0.05. *: compared with control group, p<0.05. #: compared with DMED+PBS group, p< 0.05.!: compared with DMED+ASCs^LV-control^, P<0.05).

To clarify the protective effect of ASCs^LV-shNLRP3^ on cavernous smooth muscle cells and endothelial cell function, endothelial cell markers (CD31, eNOS, and phosphorylated eNOS (P-eNOS)) and a smooth muscle marker (α-SMA) were detected by WB analysis. The protein levels of CD31, eNOS, and p-eNOS in the DMED+ASCs^LV-shNLRP3^ group were higher than those in the DMED+PBS and DMED+ASCs ^LV-control^ groups **(** **Figures 6A, B** **)**. Furthermore, the fluorescence intensity of CD31 and α-SMA in the DMED+ASCs^LV-shNLRP3^ group was much higher than that in the DMED and DMED+ASCs^LV-control^ groups and similar to that in the control group **(** **Figure 6C** **)**.

## Discussion

The desire for restorative rather than palliative management strategies for ED has stimulated interest in cell-and gene-based therapies (37). In this study, the knockdown of NLRP3 enhanced the anti-apoptotic function of and anti-ROS deposits with ASCs in a high-glucose environment, with maintenance of the paracrine function of ASCs. The knockdown of NLRP3 enhanced the post-transplantation success rate of ASCs and inhibited pyroptotic cell death of ASCs, reducing cavernous endothelial dysfunction and smooth muscle cell injury, and thereby enhancing the effects ASCs to improve DMED. However, available studies suggest that ROS can promote NLRP3 inflammasome formation, which leads to inflammation and pyroptosis (38). Therefore, the knockdown of NLRP3 might inhibit NLRP3 inflammasome-mediated pyroptosis and inflammation in ASCs in a high glucose environment. In addition, the paracrine function of stem cells participates in NLRP3-mediated cellular pyroptosis, which has been studied. For example, Liu et al (39) found that the neuroprotective effects of bone marrow mesenchymal stem cell-derived exosomes are associated with the attenuation of NLRP3 inflammasome-mediated inflammation and pyroptosis. Park et al (40) suggested that ASCs ameliorate colitis by suppressing inflammasome formation and regulating M1-macrophage populations through prostaglandin E2. Huang et al (41) showed that ASCs attenuate NLRP3 inflammasome activation and improve functional recovery after spinal cord injury through paracrine secretion. In this study, we selected three cytokines, SDF1, VEGFA and FGF2, which represent stem cell retrieval, paracrine secretion and survival, respectively.

In summary, ASCs^LV-shNLRP3^ not only enhance their own anti-apoptosis and anti-oxidative stress capacity in a high-glucose environment but also can be used to treat the disease through anti-pyroptosis and anti-inflammatory functions based on paracrine secretion.

NLRP3 acts as a sensor molecule, which occurs during self-polymerization, and it recruits ASCs and induces their assembly into large speckled structures. Subsequently, the further recruitment of caspase-1 leads to the autocatalytic activation of caspase-1. The activated caspase-1 heterodimer functions as a protease activator of the pro-inflammatory cytokines IL-1β and IL-18, as well as the soluble cell membrane protein gasdermin D. Upon proteolysis, oligomeric gasdermin N binds to membrane lipids and forms membrane pores to mediate the unconventional secretion of IL-1β and IL-18. Simultaneously, cells undergo a pro-inflammatory type of cell death known as “pyroptosis” (19). Thus, knocking down the *NLRP3* gene would potentially block pyroptosis-related signaling pathways, thereby inhibiting inflammation and thus alleviating the disease.

At the beginning of the study, we tried to explore whether pyroptosis occurs with ED in patients. However, in clinical studies, it is difficult to obtain penile tissue because of the ED. Thus, we obtained sequencing data from the public database platform the GEO database. We found DEGs related to pyroptosis in ED by using the R language. KEGG enrichment analysis found that pyroptosis-related DEGs are most frequently enriched in the NLR signaling pathway. Of them, the NLRP3/CASP1/ASC signaling pathway has been mostly researched to date (31). Further immunohistochemical analysis revealed the presence of altered NLRP3 expression in the cavernous tissue of the penis of DMED rats. This reinforces our suspicion of the possibility of pyroptosis in the cavernous tissue with DMED. We then detected IL-18, IL-1β, caspase1, GSDMD, and other pyroptosis-related molecular proteins and found that pyroptosis was indeed occurring in the cavernous body of the DMED penis.

ASCs have been used to treat DMED (27). Moreover, they can inhibit NLRP3-mediated inflammatory reactions to treat diabetes-associated complications (42). However, high glucose levels form an inflammatory environment impair the paracrine function of ASCs and threaten their survival (10, 18). LV-shNLRP3 was then used to modify ASCs. We found that ASCs^LV-shNLRP3^ were more likely to survive in a high glucose environment. Thus, our study not only demonstrated for the first time that ASCs improve diabetic erectile function by suppressing NLRP3 Inflammasome-mediated pyroptosis and inflammation in corpus cavernosum tissue but also found that LV-shNLRP3-modified ASCs exhibit a prolonged residence time in the cavernous body.

Previous studies have identified the importance of endothelial function in ED. Erection occurs with the release of nitric oxide (NO) from the vascular endothelial cells. A reduction in the endothelial cell production of NO has a negative effect on the smooth muscles in the corporal bodies and results in impaired relaxation of the smooth muscle cells with a decrease in blood supply, leading to ED. Consistent with this theory, we found that the endothelium marker CD31 (36) was reversed after treatment with ASCs^LV-shNLRP3^. In addition, e-NOS, the critical modulatory factor associated with ED, was also increased in the ASCs^LV-shNLRP3^ group as compared with levels in the DMED group. In addition, the smooth muscle contraction-associated protein α-SMA showed an altered expression pattern similar to that of CD31 in all groups. HE and Masson staining also showed that the penile corpus cavernosum tissue structure was fuller and clearer and the smooth muscle content was higher in the ASCs^LV-shNLRP3^ group than the DMED and ASCs^LV-control^ groups. Thus, the improvement in erectile function accompanies the recovery of endothelial function and the reduction in smooth muscle tissue. Meanwhile, the ICP and ICP/MAP ratio in the ASCs^LV-shNLRP3^ increased and was closer to that in the control group. Taken together, the erectile tissue recovered better after treatment with ASCs^LV-shNLRP3^ as compared to that with ASCs^LV-control^.

The limitation of this study is that the types and targets of cytokines released by ASCs need to be further explored. It is also possible that paracrine extracellular vesicles might also play a role. Further studies are thus warranted to address this. Furthermore, several other NLRs are also considered to have non-classical inflammation-independent functions and regulate a variety of signaling pathways. The modulated molecules therefore need to be identified in further studies.

## Conclusion

This study confirmed the presence of NLRP3-mediated pyroptosis in ED patients and DMED rats, which further expands our understanding of the pathogenesis of DMED. Importantly, LV-shNLRP3 enhanced the anti-apoptotic and anti-ROS functions of ASCs in a high-glucose environment, thereby enhancing their post-transplantation success rate and anti-pyroptosis function, reducing cavernous endothelial dysfunction and smooth muscle cell injury, and consequently enhancing ASCs to improve DMED.

## Data Availability Statement

The original contributions presented in the study are included in the **Supplementary Material**. Further inquiries can be directed to the corresponding authors.

## Ethics Statement

The animal study was reviewed and approved by The Animal Ethics Committee of southern Hospital of Southern Medical University, Southern Medical University.

## Author Contributions

Conceptualization, Methodology: AW. Supervision, Methodology: YP. Data curation, Writing-Original draft preparation: XZ. Experiments *in vitro* and *in vivo*: WC and FJ. Conceptualization, Design: HZ. Methodology, Writing, Editing: LC. All authors contributed to the article and approved the submitted version.

## Funding

This study was supported in part by the National Natural Science Foundation of China (82060809,82171612) and the Natural Science Foundation of Guangdong Province, China (2020A1515010114).

## Conflict of Interest

The authors declare that the research was conducted in the absence of any commercial or financial relationships that could be construed as a potential conflict of interest.

## Publisher’s Note

All claims expressed in this article are solely those of the authors and do not necessarily represent those of their affiliated organizations, or those of the publisher, the editors and the reviewers. Any product that may be evaluated in this article, or claim that may be made by its manufacturer, is not guaranteed or endorsed by the publisher.
