# Supplementary material for: NLRP3 downregulation enhances engraftment and functionality of adipose-derived stem cells to alleviate erectile dysfunction in diabetic rats
Source: Front Endocrinol (Lausanne). 2022 Jul 22;13:913296. doi: 10.3389/fendo.2022.913296 (PMC9354456; doi:10.3389/fendo.2022.913296)
Supplement: Supplementary file 3 [file Table_1.docx]

**Table S1**. Erectile dysfunction datasets (GSE10804)

| **Simple** | **Origin** | **Source name** | **Cell type** | **Used in comparison** |
| --- | --- | --- | --- | --- |
| GSM272854 | corpus cavernosum | HCCEC | HCCEC from donor with ED | ED |
| GSM272860 | corpus cavernosum | HCCEC | HCCEC from donor with ED | ED |
| GSM272861 | corpus cavernosum | HCCEC | HCCEC from donor with ED | ED |
| GSM272862 | corpus cavernosum | HCCEC | HCCEC from donor with ED | ED |
| GSM272863 | Umbilical vein | HUVEC | HUVEC from donor without ED | Non_ED |
| GSM272864 | Umbilical vein | HUVEC | HUVEC from donor without ED | Non_ED |
| GSM272865 | Umbilical vein | HUVEC | HUVEC from donor without ED | Non_ED |
| GSM272866 | Coronary artery | HCAEC | HCAEC from donor without ED | Non_ED |
| GSM272867 | Coronary artery | HCAEC | HCAEC from donor without ED | Non_ED |
| GSM272868 | Coronary artery | HCAEC | HCAEC from donor without ED | Non_ED |
| GSM272870 | Coronary artery | HCAEC | HCAEC from donor without ED | Non_ED |
| GSM272859 | corpus cavernosum | HCCEC | HCCEC from donor without ED | Non_ED |

ED: erectile dysfunction; non_ED: without erectile dysfunction
